# The contribution of PIP2-type aquaporins to photosynthetic response to increased vapour pressure deficit

**DOI:** 10.1093/jxb/erab187

**Published:** 2021-04-30

**Authors:** David Israel, Shanjida Khan, Charles R Warren, Janusz J Zwiazek, T Matthew Robson

**Affiliations:** 1 Organismal and Evolutionary Biology (OEB), Viikki Plant Science Centre (ViPS), University of Helsinki, Finland; 2 Department of Renewable Resources, University of Alberta, Canada; 3 School of Life and Environmental Sciences, University of Sydney, Australia; 4 Lancaster University, UK

**Keywords:** Aquaporin, *Arabidopsis*, CO_2_, mesophyll conductance, photosynthesis, PIP, stomatal conductance, whole-plant transpiration

## Abstract

The roles of different plasma membrane aquaporins (PIPs) in leaf-level gas exchange of *Arabidopsis thaliana* were examined using knockout mutants. Since multiple Arabidopsis PIPs are implicated in CO_2_ transport across cell membranes, we focused on identifying the effects of the knockout mutations on photosynthesis, and whether they are mediated through the control of stomatal conductance of water vapour (*g*_s_), mesophyll conductance of CO_2_ (*g*_m_), or both. We grew Arabidopsis plants in low and high humidity environments and found that the contribution of PIPs to *g*_s_ was larger under low air humidity when the evaporative demand was high, whereas any effect of a lack of PIP function was minimal under higher humidity. The *pip2;4* knockout mutant had 44% higher *g*_s_ than wild-type plants under low humidity, which in turn resulted in an increased net photosynthetic rate (*A*_net_). We also observed a 23% increase in whole-plant transpiration (*E*) for this knockout mutant. The lack of functional plasma membrane aquaporin *AtPIP2;5* did not affect *g*_s_ or *E*, but resulted in homeostasis of *g*_m_ despite changes in humidity, indicating a possible role in regulating CO_2_ membrane permeability. CO_2_ transport measurements in yeast expressing *At*PIP2;5 confirmed that this aquaporin is indeed permeable to CO_2_.

## Teaser

Plasma membrane aquaporin *At*PIP2;5 is permeable to CO_2_ and contributes to mesophyll conductance of CO_2_ in leaves.

## Introduction

Water flow across membranes, and thus through the plant, is regulated by aquaporins, which in addition to water may also conduct small neutral molecules and gases including carbon dioxide (CO_2_) and oxygen (O_2_) (Agre *et al.*, 1993; Heckwolf *et al.*, 2011; Zwiazek *et al.*, 2017). *Arabidopsis thaliana* possesses 35 different aquaporin isoforms that are divided into four subfamilies (Johanson *et al.*, 2001): plasma membrane intrinsic proteins (PIPs) located in the plasma membrane (Daniels *et al.*, 1994; Kammerloher *et al.*, 1994, 1995; Hachez *et al.*, 2014), tonoplast intrinsic proteins (TIPs) localized to the tonoplast (Maurel *et al.*, 1993; Beebo *et al.*, 2009), Nodulin26-like intrinsic proteins (NIPs) with various membrane locations (Mizutani *et al.*, 2006; Choi and Roberts, 2007), and small basic intrinsic proteins (SIPs) that are found in the membranes of the endoplasmic reticulum (Ishikawa *et al.*, 2005). PIPs are involved in a variety of processes regulating plant water flow starting from the root through the stem, as well as into and out of the leaves (Javot *et al.*, 2003; Fraysse *et al.*, 2005; Da Ines *et al.*, 2010; Ben Baaziz *et al.*, 2012; Gambetta *et al.*, 2013). Based on their phylogeny, PIPs are further divided into two subgroups, the PIP1s and PIP2s, with five and eight isoforms, respectively (Johanson *et al.*, 2001). Water permeability varies between the isoforms (Kammerloher *et al.*, 1994; Kaldenhoff *et al.*, 1995, 1998; Chaumont *et al.*, 2000; Li *et al.*, 2015) and, in fact, PIP1s are believed to transport water only when part of a heterotetramer structure also including PIP2s (Fetter *et al.*, 2004; Zelazny *et al.*, 2007; Otto *et al.*, 2010).

When a plant is provided with optimal light, water, nutrients, and temperature conditions, its rates of photosynthesis (*A*_net_) are largely determined by the rate of CO_2_ delivery to mesophyll cells, which is limited by two resistances in series: first by the rate of diffusion of CO_2_ from the leaf exterior into the intercellular airspaces through the stomata, and second by the rate of diffusion from intercellular airspaces into the chloroplast, as described by mesophyll conductance (*g*_m_). In *A. thaliana*, *At*PIP1;2 was the first aquaporin identified to be a significant contributor to *g*_m_ due to its CO_2_ permeability (Heckwolf *et al.*, 2011), but *At*PIP1;4 has now also been recognized to facilitate CO_2_ diffusion across plasma membranes (Li *et al.*, 2015). Isoforms of the PIP2 subgroup were believed to be specific to water until they were discovered to also conduct hydrogen peroxide (H_2_O_2_) (Dynowski *et al.*, 2008). However, recently, *At*PIP2;1 was also reported to conduct CO_2_ in addition to H_2_O and H_2_O_2_ (Wang *et al.*, 2016). Since all PIPs have identical selectivity filters (Wallace and Roberts, 2004), which are major determinants of substrate permeability, it is reasonable to assume that other isoforms of the PIP1 and PIP2 subgroups may also contribute to CO_2_ diffusion across the plasma membrane and affect *g*_m_ in leaves.

On the molecular scale, the structures and functions of PIPs have been reasonably well described in many plants, but this knowledge is largely limited to the cellular level, and scaling it up to the whole plant is more challenging, especially since aquaporin mutants lack an obvious phenotype under low evaporative demand conditions commonly used in Arabidopsis research. Our main aim was to determine the respective roles of three Arabidopsis PIP isoforms (*At*PIP2;2, *At*PIP2;4, and *At*PIP2;5) in the regulation of stomatal conductance (*g*_s_) and *g*_m_, both of which can substantially limit rates of photosynthesis. At saturating light, the CO_2_ concentration drops to about half that of atmospheric (*c*_a_) at the sites of carboxylation (*c*_c_). The drawdown from *c*_a_ to *c*_i_ (intercellular CO_2_ concentration) is restricted by *g*_s_, accounting for ~60% of the limitation in CO_2_ diffusion, while *g*_m_ accounts for the remaining 40%. Therefore, *g*_m_ is a large, but still poorly understood, limitation to photosynthesis. Soil water deficit has similar effects on *g*_s_ and *g*_m_ (Warren, 2008*a*, b), indicating that they are interconnected and, at least in part, controlled by the same mechanisms. Furthermore, since most of the resistance to the diffusion of CO_2_ within the leaf comes from the liquid phase (Warren, 2008*b*), it is conceivable that *g*_m_ could be regulated through the activity of aquaporins. Thus, PIPs may be instrumental in modulating the link between *g*_s_ and *g*_m_. Ultimately, modulating PIP function to increase *g*_m_ without altering *g*_s_ would enhance *A*_net_ without an accompanying increase in transpiration, and improve plant water use efficiency.

Past studies of the functions of aquaporins have generated a wealth of information concerning single isoforms in Arabidopsis, maize, and various other herbaceous and woody plant species (Fetter *et al.*, 2004; Zelazny *et al.*, 2007; Ben Baaziz *et al.*, 2012; Gambetta *et al.*, 2013; Li *et al.*, 2015). However, the multitude of different species and methods employed also makes it difficult to develop a cohesive picture of the roles of aquaporins in plants. In this study, we compared three different single knockout mutants of *A. thaliana* and their wild type (WT) to clarify their putative roles in whole-plant function. Assigning more clearly defined and specific roles to the different isoforms will aid in determining whether there is redundancy among plant aquaporins. An indication that different aquaporins are not redundant is given by their differing expression patterns. In adult plants, *AtPIP2;2* is highly expressed throughout the plant (Javot *et al.*, 2003), while *AtPIP2;5* reaches moderate to high levels of expression in mature leaves and guard cells, respectively (Alexandersson *et al.*, 2010). However, its expression levels are lower in roots (Alexandersson *et al.*, 2005), while *AtPIP2;4* is only moderately expressed in leaves but highly expressed in roots (Javot *et al.*, 2003). Since the function of PIPs also depends on their mutual interactions in the tetramer structure (Fetter *et al.*, 2004; Zelazny *et al.*, 2007; Otto *et al.*, 2010), we furthermore examined two double mutants (*pip2;2x2;4* and *pip2;4x2;5*) as well as a triple mutant (*pip2;2x2;4x2;5*).

In the present study, we grew plants in two environments differing in their ambient relative humidity, to compare plant responses to different vapour pressure deficits (Vpds). A high Vpd promotes high rates of transpiration and thus triggers plant responses aimed at conserving water such as stomatal closure. Earlier studies failed to find a visible phenotype for aquaporin knockout mutants growing under ideal (low Vpd) conditions (Javot *et al.*, 2003; Da Ines *et al.*, 2010; Heckwolf *et al.*, 2011; Wang *et al.*, 2016), so this high Vpd treatment was applied to increase the relative contribution of aquaporins to plant water flow and the likelihood of producing a distinct phenotypic response. We therefore hypothesize that PIP2 knockout mutants would produce a phenotypic response under conditions of high Vpd, which, under low Vpd, would go unnoticed or show as statistically non-significant trends only.

## Materials and methods

### Plant material

T-DNA single knockout mutants of *A. thaliana* were obtained from the Nottingham Arabidopsis Stock Centre (NASC; www.arabidopsis.org) for the following aquaporin grnes: *PIP2;2* (N871747), *PIP2;4* (N105980), and *PIP2;5* (N117303) in the Columbia background (Alonso *et al.*, 2003). All genotypes were checked by PCR to confirm the correct T-DNA insertion and homozygosity. Only homozygous plants were used to grow a seed stock and in the subsequent experiments. Multiple knockout plants were created by crossing, which resulted in a total of two different double mutant lines—*pip2;2x2;4*, *pip2;4x2;5*—and one triple mutant—*pip2;2x2;4x2;5*. It was not possible for us to create the *pip2;2x2;5* mutant for this study.

Since *g*_m_ acclimates to the environmental conditions (Warren, 2008*b*), we grew the plants under different humidities *in situ* in the two environments differing in Vpd instead of raising them in the same conditions and subjecting them to short-term treatments, which would probably affect both *g*_m_ and *g*_s_ and thus prevent us from separating the role of aquaporins in these two processes. The environmental parameters for the high humidity and low humidity conditions are summarized in Supplementary Table S1.

### Low humidity growing conditions

The experiment was carried out in a greenhouse at the University of Sydney, Sydney, Australia. Seeds of the *A. thaliana* genotypes were sown in pots containing a pre-fertilized potting mix (Scotts Osmocote, plus trace elements) and germinated in the light under the conditions described in Supplementary Table S1. Seedlings were transplanted 4 d after germination into 700 ml pots. Pots were overfilled with the same potting mix, as described in Flexas *et al.* (2007b). Young seedlings were kept under a transparent plastic cover for several days after transplanting to keep them moist. The pots were arranged randomly and rearranged every second day to ensure even light interception and to minimize any effects of air movement caused by the air conditioning. All measurements were carried out between 09.30 h and 16.30 h on 25- to 39-day-old plants using only fully expanded leaves at least 4 cm long. *A*_net_ is reported to be stable over this time period (Flexas *et al.*, 2007b). The experiment was conducted during the Australian spring from mid-October to mid-December 2015. During this time, new seeds were planted at weekly intervals to continuously provide plants of equivalent ages for measurements.

Plants were provided with ample water in order to prevent soil water stress signalling from the roots, since our aim was to expose plants to low air humidity without imposing other accompanying stresses. Water was provided from below as soon as the soil surface had dried (every 2–3 d), but after the day’s measurements.

### High humidity growing conditions

This part of the experiment was carried out in a growth room at the University of Helsinki, Helsinki, Finland. Seeds of the *A. thaliana* genotypes were sown and grown in a pre-fertilized peat–vermiculite mixture (1:1) in 350 ml pots. Seedlings were transplanted 4 d after germination into overfilled pots as described above. Plants were grown in a growth chamber under conditions described in Supplementary Table S1. Measurements were carried out between 09.00 h and 16.00 h from October 2016 until January 2017 on 27- to 33-day-old plants using only fully expanded leaves at least 4 cm long. The plants were watered as in the low humidity treatment described above; however, due to the higher air humidity in these growing conditions, watering was required only once a week.

### Gas exchange measurements

All gas exchange measurements were carried out with the portable photosynthesis system LI6400XT infrared gas analyser equipped with a fluorescence chamber (LI-COR Biosciences, Lincoln, NE, USA) during the same 7 h time window every day. The leaf chamber (leaf chamber fluorometer), a 2 cm^2^ circular cuvette, allowed single leaves to be measured.

Photosynthetic light responses were measured under non-photorespiratory conditions in the low air humidity (LH) treatment (1% O_2_, 400 µmol mol^–1^ CO_2_) to determine the relationship between photosynthetic rate and light intensity as well as the rate of electron transport (*J*). High purity N_2_ gas (BOC Gas, Australia) was mixed with air to create a 1% O_2_ mixture, directly attached to the air inlet of the LICOR-6400. Under non-photorespiratory conditions, *J* is entirely dependent on gross photosynthesis *A* (Warren and Dreyer, 2006):


$$\begin{array}{l} J=4\left(A+R_{d}\right) \end{array}$$ (1)

where *R*_d_ is the respiration in the light. The curves were measured using an automated program with the following fixed settings: temperature 25 °C; leaf fan, fast; reference CO_2_ concentration (CO_2_R), 400 µmol mol^–1^; flow, 300 µmol s^–1^; and 10% blue light. Light adaptation lasted for 30 min at maximum irradiance [photosynthetically active radiation (PAR) 2000 µmol m^–2^ s^–1^]. The light curves began at the highest irradiance and decreased at ~4 min intervals of acclimation time between each step: 2000, 1500, 1000, 500, 200, 100, 50, 20, and 0 µmol m^–2^ s^–1^. Light curves measured under high air humidity (HH) were measured using the same automated program, but under ambient O_2_ concentrations.

Full *A*–*c*_i_ curves were measured in the high humidity growth chamber experiment to determine whether the standard CO_2_ concentration used in our gas exchange measurements falls within the CO_2_-limiting range for all plant lines. An automated program was used throughout the curve with the same settings as for the light curves except a constant PAR of 1500 µmol m^–2^ s^–1^. Plants acclimated for 10 min in the leaf chamber before the first measurement at 400 µmol mol^–1^ CO_2_. After the first measurement, the following steps with 3 min acclimation time between each step were used to obtain a complete *A*–*c*_i_ curve: 450, 550, 650, 750, 850, 1000, 1500, 2000, 400, 350, 300, 250, 200, 150, 100, and 50 µmol mol^–1^ (Warren and Dreyer, 2006).

The Laisk method (Laisk, 1977; Brooks and Farquhar, 1985) was used to estimate *c*_i_* (photorespiratory compensation point) and *R*_d_. In both experiments, LH and HH conditions, an automated program with the same settings was used for the Laisk method: temperature, 25 °C; leaf fan, fast; CO_2_R, 400 µmol mol^–1^; flow, 200 µmol s^–1^; PAR, 1500 µmol m^–2^ s^–1^; and 10% blue light. The *A*–*c*_i_ curves were measured at PAR 300, 150, 100, and 50 µmol m^–2^ s^–1^ and, after each curve, CO_2_ was returned to 400 µmol mol^–1^ for 5 min to maintain Rubisco activation. The CO_2_ steps used for the curve were 150, 125, 100, 75, and 50 µmol mol^–1^ with ~3 min acclimation time between each step.

**Fig. 1.**
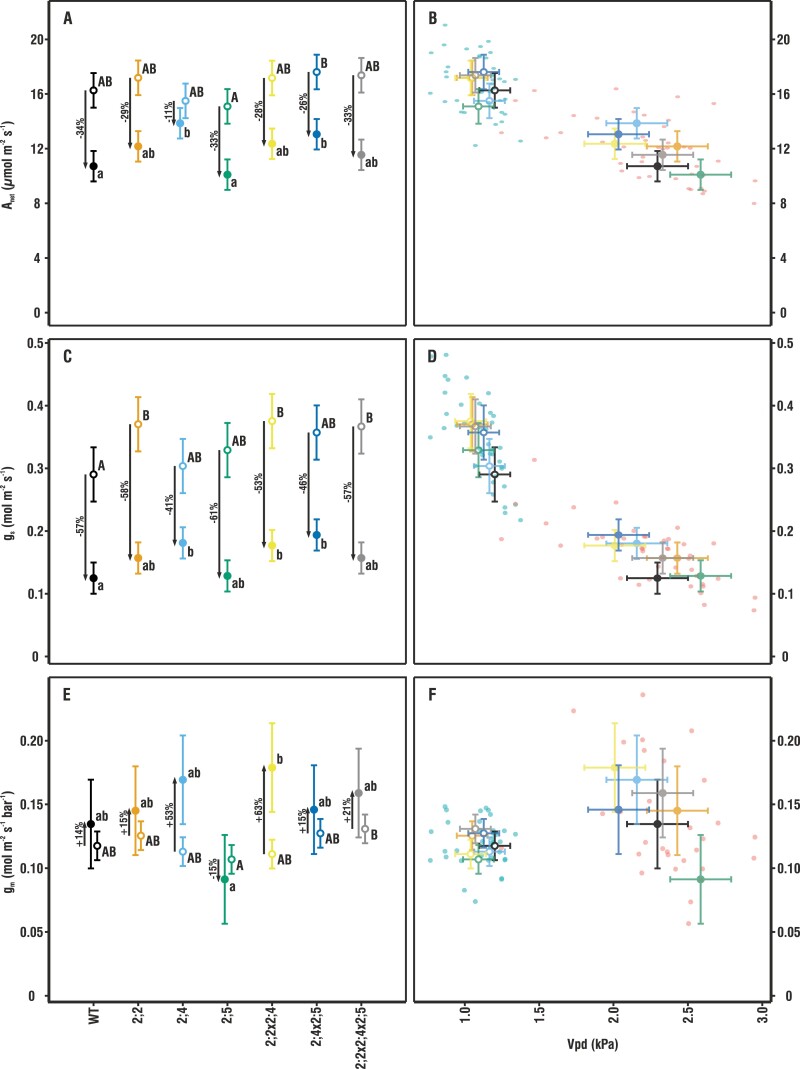
Comparison of gas exchange for plants grown under low and high humidity. The left-hand panels compare mean ±pooled SE of *A*_net_ (A), *g*_s_ (C), and *g*_m_ (*E*) among genotypes (*n* = 4–9). Upper and lower case letters indicate statistically significant differences between means within the high (HH; open circles) and low humidity (LH; filled circles) treatments, respectively. The right-hand scatter plots give individual plant measurements as well as genotype means for gas exchange with respect to cuvette Vpd; *A*_net_ (B), *g*_s_ (D), and *g*_m_ (F). There were no significant differences in the relationship to cuvette Vpd among the genotypes within the HH and LH set. Gas exchange parameters are summarized in Supplementary Table S2.

*g*_min_ describes the minimum conductance; that is, the rate of water loss from leaves due to direct diffusion through the cuticle and leaky stomata. This pathway for gas exchange is often neglected as it represents only values in the range of 5–10 mmol m^–2^ s^–1^ H_2_O (Fig. 3B; Supplementary Table S2; Duursma *et al.*, 2019) as compared with a *g*_s_ of 100–400 mmol m^–2^ s^–1^ for actively transpiring Arabidopsis leaves (Fig. 1C). Nevertheless, all gas exchange data were adjusted to account for *g*_min_ as well as any CO_2_ leaks into or out of the LICOR measuring chamber. We estimated *g*_min_ for fully expanded leaves using the protocol described by Sack and Scoffoni (2010) with some modifications to accommodate fragile and small Arabidopsis leaves: for each data point, three leaves that were suitably large for gas exchange measurements were excised from 21 plants per line close to the centre of the rosette. The leaves were weighed for initial FW, placed flat on millimetre graph paper, and photographed to calculate their initial leaf area using ImageJ as described by Wang (2016). After photographing, they were placed on labelled Petri dishes and allowed to dry at room temperature (20 °C) for 1 h until complete stomatal closure. From this point on, leaves were weighed every 25–30 min. The first time point after the 1 h drying period was taken to be time 0. After 6–10 time points had been obtained over the course of 3–4 h, the leaves were again photographed to calculate their final leaf area. The collected data were input into the ‘*g*_min_ Analysis Spreadsheet Tool’ (Sack and Scoffoni, 2010) in order to calculate *g*_min_.

The same leaves that had been used for estimating *g*_min_ were floated on water overnight to obtain their saturated weight (SW). Finally, the leaves were dried in a drying oven overnight at 60 °C to obtain their DW. The relative water content (RWC) was then calculated using the following formula:


$$\begin{array}{l} RWC=100*\frac{FW-DW}{SW-DW} \end{array}$$ (2)

The leak flow was calculated using the manufacturer’s instructions; however, we placed an intact leaf in the chamber instead of carrying out the estimation for an empty chamber. In the dark, the leaf’s respiration rates should not be affected by changing CO_2_ concentrations or flow rates and thus can be considered constant. Therefore, we were able to obtain a diffusion coefficient of 0.40 mol s^–1^ for the actual measuring conditions, which is similar to the 0.44 mol s^–1^ obtained by Flexas *et al.* (2007a).

The mesophyll conductance of CO_2_ was estimated using the variable *J* method as described by Harley *et al.* (1992):


$$\begin{array}{l} g_{m}=\;\frac{A}{c_{i}-\;\frac{{c_{i}}^{*}\left[\;J+8\;\left(A+\;R_{d}\right)\right]}{J-4\;\left(A+\;R_{d}\right)}} \end{array}$$ (3)

The variables *A*, *c*_i_, and *J* were obtained from the combined gas exchange and chlorophyll fluorescence measurements. *R*_d_ as well as *c*_i_* were estimated from the Laisk method using slope–intercept regression (Walker and Ort, 2015). One of the largest sources of error when using the variable *J* method to estimate *g*_m_ can be the mismatch between the measurement of electron transport rates and photosynthesis (Flexas *et al.*, 2013), however, for thin Arabidopsis leaves, this discrepancy is relatively small. For each estimate of *g*_m_, we also calculated the δ*C*_c_/δ*A* as described in Harley *et al.* (1992) and excluded measurements that fell outside the recommended range of 10 <δ*C*_c_/δ*A* <50.


$$\begin{array}{l} \frac{\delta C_{c}}{\delta A}=\frac{12{c_{i}}^{*}J}{{\left[J-4\left(A+R_{d}\right)\right]}^{2}} \end{array}$$ (4)

As we did not find any statistically significant differences in either R_d_ or *c*_i_* between the different genotypes, we calculated a global average for both variables (*R*_d_=0.914±0.031 µmol m^–2^ s^–1^, *n*=69; *c*_i_*=38.95±0.70 µmol mol^–1^, *n*=65) in order to obtain a more robust estimate of *g*_m_. In comparison, Walker and Ort (2015) reported values of *R*_d_=1.04±0.33 µmol m^–2^ s^–1^, *c*_i_*=40.0±0.7 µmol mol^–1^ for *Nicotiana tabacum* and *R*_d_=1.02±0.22 µmol m^–2^ s^–1^, *c*_i_*=40.8±2.4 µmol mol^–1^ for *Glycine max*.

The average *g*_m_ obtained for each mutant line was used to calculate *A*–*c*_c_ curves from the previously measured *A*–*c*_i_ curves. Parameters *J*_max_ (maximum rate of electron transport), *V*_cmax_ (maximum rate of carboxylation), and the inflection point shown in Supplementary Table S2 were estimated from fitted *A*–*c*_c_ curves utilizing the ‘Plantecophys’ R package version 1.4.4 (Duursma, 2015) using the chloroplastic Michaelis–Menten constants for CO_2_ (*K*_c_) and O_2_ (*K*_o_) at *c*_c_ as described by Bernacchi *et al.* (2002), which allowed us to calculate *K*_m_=620.3322 to be used in the curve fit. The fitted *A*–*c*_c_ curves were not normalized to 25 °C as the measurements were done at this temperature and the *R*_d_ as well as *c*_i_* estimates presented above were provided to obtain a more accurate fit. Using an orthogonal non-linear least-squares regression (‘onls’ package in R), the following parameters were extracted from the light curves: saturating photosynthesis (*A*_sat_), quantum efficiency (φ), *R*_d_, degree of curvature between the light-limited and CO_2_-limited part of the curve (θ), light compensation point (LCP), and irradiance at 75% *A*_sat_ (Supplementary Table S2).

### Whole-plant transpiration measurements

We estimated the water transpired by entire plants under the LH and HH growing conditions over a 30 d period. This experiment at the University of Helsinki used a Fitoclima 1200 growth chamber (Aralab, Rio de Mouro, Portugal) to create the LH and HH growing conditions (day length, temperature, humidity, etc.). The air circulation inside the chambers was set to maximum to ensure adequate ventilation and a high boundary layer conductance. A total of 10–16 plants of each genotype were planted in 350 ml pots which had their soil surface sealed with a plastic membrane preventing evaporative water loss from the pot. A small hole was cut into the membrane through which the plants grew. Prior to transplanting, the pots were saturated with water to their water-holding capacity (52% gravimetric soil moisture content in this case). Immediately after transplanting, the pots were weighed with the plastic cover and the plant in order to obtain the initial weight (W_i_). Thereafter, pots were weighed roughly every 2 d and, if necessary, a known volume of water was added to the soil surface using a syringe. Soil moisture was maintained at a minimum of 25% (gravimetric), ensuring the soil surface never dried up completely. Total leaf area was measured weekly for each plant using ImageJ as described by Wang (2016) and, at the end of the experiment, plant FW was measured. The total amount of water transpired by each plant was calculated with respect to cumulative leaf area using the following formula:


$$\begin{array}{l} E=\frac{V+\left(W_{i}-W_{f}-FW\right)}{A_{t}} \end{array}$$ (5)

where V denotes the volume of water added to one pot over the course of experiment, W_i_ is the initial weight of the pot including the plant, W_f_ the final weight of the pot including the plant at the end of the experiment, FW is the fresh weight of the plant at the end of the experiment, and A_t_ is the total cumulative leaf area of the plant over the course of 30 d.

### RNA extraction and cloning

Tissue from 6-day-old seedlings of *A. thaliana* ecotype Columbia-0 were used for RNA extraction. Total RNA was extracted using the QIAGEN RNeasy Plant Mini Kit (Qiagen, Valencia, CA, USA). RNA concentration and purity were assessed using the Thermo Scientific™ NanoDrop™ One Microvolume UV-Vis Spectrophotometer (Thermo Scientific, Waltham, MA, USA). The first-strand cDNAs were synthesized from 1 μg of total RNA using Superscript II reverse transcriptase (Invitrogen, Carlsbad, CA, USA) and an oligo(dT) primer. Coding sequences of Arabidopsis *PIP2;5* (AT3G54820) and carbonic anhydrase 1 (*CA1*; AT3G01500) were amplified with Phusion DNA Polymerase using the primers listed in Supplementary Table S4. The PCR products of the expected size were eluted from the gel and purified using the Wizard® SV Gel and PCR Clean-Up System (Promega, Madison, WI, USA). The PCR products were then cloned into a pCR2.1-TOPO vector using the Topo TA Cloning kit (Invitrogen) and transformed into DH5α chemically competent cells (Invitrogen). About 3–6 white colonies were sequenced for each PCR product using M13 sequencing primers.

### Plasmid construction and yeast transformation

The ORF of *AtPIP2;5* and carbonic anhydrase 1 (*AtCA1*) were cloned as Gateway entry clones in plasmid pDONR221 (Invitrogen). ORFs of *AtPIP2;5* and *CA1* from the entry clones were shuttled into the galactose-inducible yeast expression plasmid pAG426GAL-ccdB (Addgene: Plasmid #1415) and pAG425GAL-ccdB (Addgene: Plasmid #14153), respectively, by Gateway LR cloning reaction. The S.*c.* EasyComp™ Transformation Kit (ThermoFisher Scientific) was used to transform the *Saccharomyces cerevisiae* yeast strain (INVSc1 from ThermoFisher Scientific) with each plasmid DNA. Double transformants used for CO_2_ permeability measurements containing *AtCA1* and *AtPIP2;5* (AtCA1::AtPIP2;5) constructs were selected by ura3 and leu2 complementation. Expression of the constructs was verified by quantitative real-time PCR (qRT-PCR) using SYBR Green I dye in an Applied Biosystems 7500 Fast system.

Subsequently, the subcellular localization of *At*PIP2;5 in yeast cells was investigated using fluorescence microscopy (Supplementary Fig. S2). The attB-PCR fragment of *At*PIP2;5 was transferred into pDONR221 with BP clonase and shuttled from the entry clones into the yeast expression plasmid pAG426Gal-ccdB-eGFP by Gateway LR cloning reaction to generate C-terminally tagged protein–enhanced green fluorescent protein (eGFP) fusions. Yeast transformation was carried out as described above. Single colony isolates of the yeast strains were grown to midlog phase in 2% Glu/−Ura medium at 30 °C. Cultures were spun down and re-suspended in the same volume of 2% Gal/−Ura to induce expression of the constructs.

The yeast strains expressing pAG426Gal::At2;5, pAG425Gal::AtCA, and PAG426Gal::At2;5XpAG425Gal::AtCA were induced by changing the carbon source of the medium from glucose to galactose for 24 h (1.2 *g*, 30 °C). RT-PCR was performed to determine the relative abundance of mRNA in the yeast strains. An RNeasy Mini Kit (QIAGEN, Venlo, Limburg, The Netherlands) was used to extract total RNA from the yeast cells according to the manufacturer’s instructions. The QuantiTect Reverse Transcription Kit (Qiagen) was used to synthesize first-strand cDNAs with 500 µg of total RNA, according to the manufacturer’s instructions, and the qRT-PCR was performed as above. The relative expression of all genes was calculated using the ΔΔCt method with actin as a reference gene (Supplementary Fig. S3).

### CO_2_ transport measurements

The entry of CO_2_ through the plasma membrane was followed by intracellular acidification and decreased fluorescence in whole yeast cells loaded with fluorescein diacetate. Loading of the yeast cells was carried out according to the protocol described by Bertl and Kaldenhoff (2007). In brief, cells were harvested by centrifugation and then resuspended in loading buffer (50 mM HEPES-NaOH pH 7.0, 5 mM 2-deoxy-d-glucose) with 50 μM fluorescein diacetate, incubated for 14 min at 30 °C, shaken at ~225 rpm, and centrifuged again at 1700 *g* for 3 min at 4 °C. The cells were then resuspended in incubation buffer (25 mM HEPES. 75 mM NaCl) and kept on ice until use. Before use, the cells were resuspended in incubation buffer to a final OD_600_ of 60, after which 50 μl were dissolved in the incubation buffer and mixed rapidly in a 1:1 (v/v) ratio with CO_2_-Mixing Buffer (25 mM HEPES, 75 mM NaHCO_3_, pH 6) at a rate of 100 μl s^–1^ in a stopped flow spectrophotometer (Applied Photophysics, DX.17 MV). Entry of CO_2_ into yeast cells expressing *At*CA1 resulted in H_2_CO_3_^–^ formation (and subsequent dissociation into H^+^ and CO_3_^–^) and thus intracellular acidification, as indicated by a decrease in fluorescence intensity. The spectrophotometer emitted at a λ of 490 nm (maximum excitation wavelength of the fluorescein). The receiver had a filter attached that did not allow the passage of wavelengths below 515 nm, because fluorescein emits at a λ of no longer than 514 nm. The fluorescence was recorded over time and the conductivity quantification (K-relative) was calculated by fitting the experimental data to a decreasing exponential function during the first 8.0 ms using SigmaPlot 11.0 (Systat Software Inc., Chicago, IL, USA).

### Gene expression analysis

Transcript abundance was measured by qRT-PCR for each plant line and growing condition. Under LH, 4–6 rosettes/genotype, and under HH 12 rosettes/genotype were harvested and immediately frozen in liquid nitrogen. Due to the larger sample number under HH, we combined three samples and treated them together, resulting in *n*=4 for HH. RNA was extracted using the GeneJET Plant RNA Purification Mini Kit (ThermoFisher Scientific) according to the manufacturer’s instructions, with the exception that the Plant RNA Lysis Solution was supplemented with β-mercaptoethanol instead of DTT. The quality and concentration of the extracted RNA were determined with an ND-1000 Spectrophotometer (ThermoFisher Scientific), and 1 µg of RNA was used for cDNA synthesis following DNase I treatment. Maxima H Minus Reverse Transcriptase, oligo(dT)19, and dNTP (ThermoFisher Scientific) were used in a 30 µl reaction volume for cDNA synthesis, which was then diluted to a final volume of 70 µl. A 1 μl aliquot of cDNA was used for PCR in triplicate with 5× HOT FIREPol EvaGreen qPCR Mix Plus (Solis BioDyne, Tartu, Estonia) with a CFX 384 Real-Time PCR detection system (Bio-Rad, Hercules, CA, USA) in triplicate. PIP-specific primers were taken from Alexandersson *et al.* (2010) and information on reference gene primers can be found in Supplementary Table S4. Ct values were converted using the ΔΔCt method employing all three reference genes listed in Supplementary Table S4 and ln-transformed for statistical analysis.

### Data processing and statistical analysis

ANOVAs were conducted separately for the LH and HH experiment in R (package Deducer) using a linear model with plant genotype and the measured variable as the factors, and for each graph/panel we calculated the pooled standard error except for gene expression data. For estimations of *g*_min_ and RWC, *n* = 21; for all gas exchange measurements, *n*=3–9; for whole-plant transpiration, *n*=10–16; while for gene expression, *n*=4–12. Tukey’s multiple comparison was used to compare the means of all measured variables for the mutant lines with the WT as well as with each other.

Generalized additive mixed modelling (GAMM, package=‘mgcv’) (Wood, 2017) was used to evaluate the photosynthetic response curves, with the mutant line as a parametric term and a smoothing term for PAR and *c*_i_. Due to heterogeneous variation, we employed the weighting function ‘weights=varExp’. The fluorescence decay rates from the stopped-flow measurements were compared using Duncan’s multiple range test.

## Results

### Effects of PIP2 aquaporins on gas exchange under high and low humidity

The difference in evaporative demand between the HH and LH growing conditions had a clear effect on gas exchange and was most strikingly visible in the values of *g*_s_ and its consequences for *A*_net_ (Fig. 1A–D).

In the LH environment, all plant genotypes displayed 41–61% lower *g*_s_ than in the HH environment (Fig. 1C, D). However, single and double mutant plants lacking *At*PIP2;4 decreased their stomatal conductance less than the WT in response to LH. The stomatal conductance of the *pip2;4*, *pip2;2x2;4*, and *pip2;2x2;4x2;5* mutants was significantly higher than that of the WT under LH, but nevertheless reduced compared with the HH treatment (41, 53, and 57% for *pip2;4*, *pip2;2x2;4*, and *pip2;2x2;4x2;5*, respectively). The WT *g*_s_ under LH was reduced by 57% compared with HH.

In the HH environment, there was a tendency for the genotypes lacking *At*PIP2;2 to have higher values of *g*_s_ compared with the WT (*pip2;2*, *P*=0.089; *pip2;2x2;4*, *P*=0.055; and *pip2;2x2;4x2;5*, *P*=0.091). Although for the individual mutants alone this was not a statistically significant effect at *P*<0.05, when all mutant plants lacking *At*PIP2;2 were considered together as a group, the increase of 28% compared with the WT was statistically significant.

Those genotypes in which Vpd had a large effect on *g*_s_ also displayed large differences in *A*_net_ (Fig. 1A–D; Supplementary Table S2). In the WT, *A*_net_ was reduced by 34% in the LH environment compared with the HH environment, whereas the reduction was only 11% for *pip2;4*. Rates of photosynthesis were very uniform among all genotypes under the HH condition (Fig. 1A, B). Under LH, however, *A*_net_ declined less in *pip2;4* and *pip2;4x2;5* than in the WT, as would be expected given their smaller decrease in *g*_s_ from HH to LH compared with the WT. In addition, the *pip2;5* single mutant had lower values of *A*_net_ than the *pip2;4x2;5* double mutant under both growing conditions.

The *g*_m_ of the mutant plants did not significantly differ from that of the WT under either LH or HH conditions (Fig. 1E). However, unlike *A*_net_ or *g*_s_, *g*_m_ increased under LH in all genotypes except *pip2;5*. Pairwise comparisons revealed that *pip2;5* tended to have a lower *g*_m_ than *pip2;4x2;5* and *pip2;2x2;4x2;5* under HH (Fig. 1E, F). Similarly, under LH, *pip2;5* had marginally lower *g*_m_ compared with *pip2;4*, *pip2;2x2;4*, and *pip2;2x2;4x2;5* (Fig. 1E, F). There was a similar pattern of typically lower *A*_net_ in *pip2;5* versus *pip2;4x2;5* or *pip2;2x2;4x2;5* (Fig. 1A).

The Vpd in the cuvette during measurements differed ~2-fold in LH versus HH, affecting both *A*_net_ and *g*_s_, with individual plants, as well as means for each genotype, forming two distinct clusters (Fig. 1B, D, F). Values of *g*_m_ differed less, but nevertheless also formed two distinct clusters. Variation in cuvette Vpd was larger under LH (2.0–2.5 kPa) than under HH (1.0–1.25 kPa), but none of the genotypes differed significantly from the WT under either growing condition (Supplementary Table S2), and thus Vpd does not account for statistically significant differences in *A*_net_, *g*_s_, or *g*_m_ among genotypes.

### Whole-plant transpiration

**Fig. 2.**
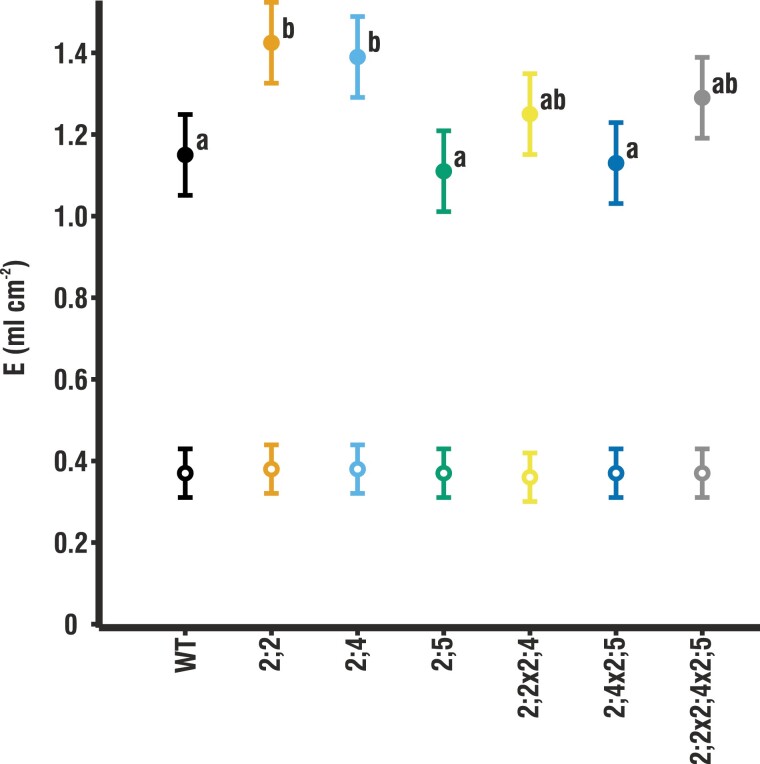
Whole-plant transpiration in terms of cumulative leaf area under low and high humidity. Transpiration is shown under low humidity (LH; filled circles) and high humidity (HH; open circles) over the course of 30 d (*n*=10–16). Given are means ±pooled SE. Whole-plant transpiration data as well as the SE of each individual genotype are shown in Supplementary Table S2. Letters indicate statistically significant differences between mutants. No significant differences were found under HH.

Whole-plant transpiration (*E*) was measured over the course of 30 d beginning from germination to determine whether genotypic variation in instantaneous leaf-level gas exchange measurements scaled to long-term whole-plant differences in water use. Despite the higher *g*_s_ (Fig. 1C) under HH growing conditions, *E* was low and did not differ among genotypes (Fig. 2). Under LH conditions, *E* was 3–4 times higher, with *E* of *pip2;2* and *pip2;4* significantly higher than that of the WT, which is in line with their increased *g*_s_ under the same LH growing conditions. Despite similar values of *g*_s_ (Fig. 1C), *pip2;2x2;4* and *pip2;4x2;5* did not differ significantly from the WT in their whole-plant transpiration.

### Effects of PIP2 aquaporins on mesophyll conductance under high and low humidity measured through *A*–response curves

**Fig. 3.**
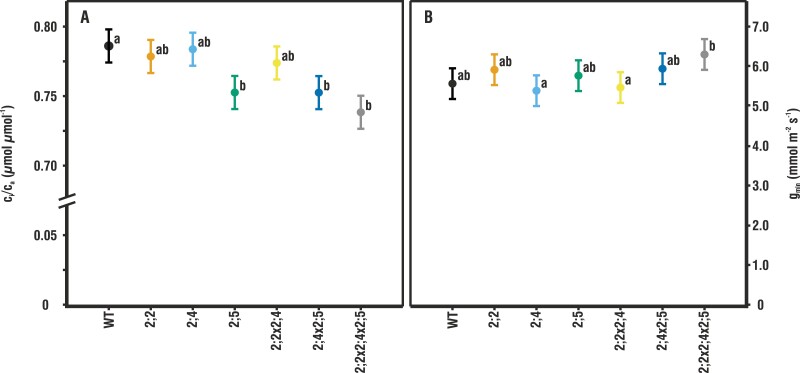
*c*_i_/*c*_a_ and *g*_min_ in the wild type (WT) and mutants. (A) The mean ±pooled SE *c*_i_/*c*_a_ for measurement points of the *A*–*c*_i_ curves at higher than ambient CO_2_ concentrations (≥400 µmol mol^–1^ air) and under high humidity (*n*=8–10). Letters indicate statistically significant differences compared with the WT. (B) The mean ±pooled SE for minimum conductance, *g*_min_, under high humidity (*n* = 21). Letters indicate statistically significant differences between mutants.

Using the mean *g*_m_ for each mutant line, we calculated *A*–*c*_c_ curves (Supplementary Fig. S1) from the measured *A*–*c*_i_ curves to detect effects of the PIP2 mutations on the limits of CO_2_ fixation. There were no significant differences among the genotypes for either the *J*_max_ or *V*_cmax_ (Supplementary Table S2). Furthermore, there were no differences in *c*_i_/*c*_a_ at low CO_2_ (<400 µmol CO_2_ mol^–1^ air), but at high CO_2_ (>400 µmol CO_2_ mol^–1^ air), *c*_i_/*c*_a_ was significantly lower in all mutants lacking *At*PIP2;5 than in the WT (Fig. 3A).

**Fig. 4.**
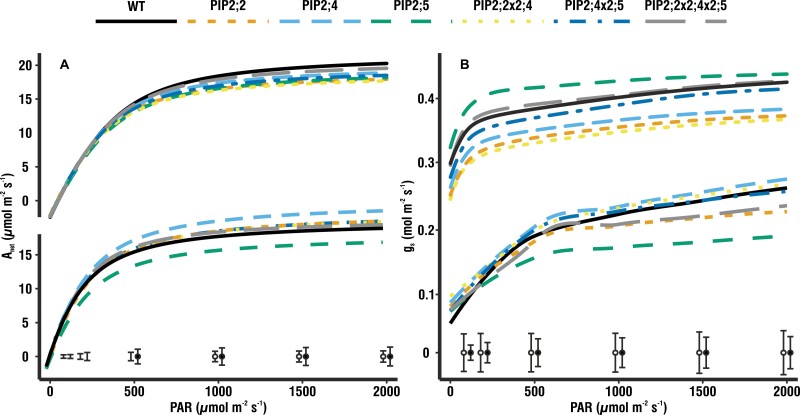
Light–response curves. (A) Light–response curves measured under high humidity (HH; above, *n*=9–11) and low humidity (LH; below, *n*=4–7) showing the rate of photosynthesis in response to increasing radiation. *A*_sat_ reaches similar values under both growing conditions. Under LH, the *pip2;5* mutant stands out, displaying 13% lower *A*_net_ compared with the wild type (WT). *Pip2;4* showed the opposite trend, with 21% higher *A*_net_ compared with the WT. (B) Light–response curves measured under HH (above, *n*=9–11) and LH (below, *n*=4–7) showing *g*_s_ in response to increasing radiation. Under HH, *g*_s_ is clearly higher and much more stable over the entire range of irradiances. Under LH, *pip2;5* displayed a much slower and 5% smaller response of *g*_s_ compared with the WT. *Pip2;4* showed 19% higher *g*_s_ compared with the WT. Fitted means with the pooled SE at each measuring point under HH (open circles) and LH (filled circles) are given at the bottom of the graph.

In line with the steady-state measurements of gas exchange, *pip2;5* produced light–response curves with lower values of *A*_net_, as well as *g*_s_, than the other genotypes (13% and 5% lower, respectively, compared with the WT; Fig. 4). At the points on the curve at saturating PAR (500–2000 µmol m^–2^ s^–1^ for these plants), *A*_net_ was similar in the WT plants and the examined genotypes (Fig. 4). However, for measurement points at subsaturating PAR (<500 µmol m^–2^ s^–1^), *A*_net_ of *pip2;5* was lower than that of all other genotypes including the WT (21–50% lower), which resulted in a significantly lower quantum efficiency, φ. At low light intensities, *g*_s_ did not differ among genotypes, whereas at higher light intensities (1000–2000 µmol m^–2^ s^–1^), *pip2;5* displayed significantly lower *g*_s_ compared with all other genotypes including the WT. In addition to the traditional parameters extracted from photosynthetic light–response curves, we also used GAMM which enabled us to also analyse the mutants’ stomatal response to changing light intensities. These analyses confirmed the differences in the shape of light–response curves, which were significant for *A*_net_, corroborating the significant difference we report in φ, as well as *g*_s_ in *pip2;5* compared with the WT. Furthermore, the curves for *pip2;2* and *pip2;2x2;4x2;5* also differed significantly from the WT in the response of *g*_s_ to increasing PAR.

The light–response curves measured under HH (Fig. 4 top) were also in line with the steady-state gas exchange measurements and did not produce significant differences between the mutants and the WT, with the exception of *A*_sat_, which was lower for *pip2;2*, *pip2;2x2;4*, and *pip2;4x2;5*.

Values of the minimum conductance of water (*g*_min_) (Fig. 3B) were within the expected range of 5–10 mmol m^–2^ s^–1^. No significant differences in *g*_min_ were found between the WT and any of the mutants. Only *g*_min_ of *pip2;2x2;4x2;5* tended to be higher than the WT, and higher than *pip2;4* and *pip2;2x2;4*.

### CO_2_ conductance of AtPIP2;5 expressed in yeast

**Fig. 5.**
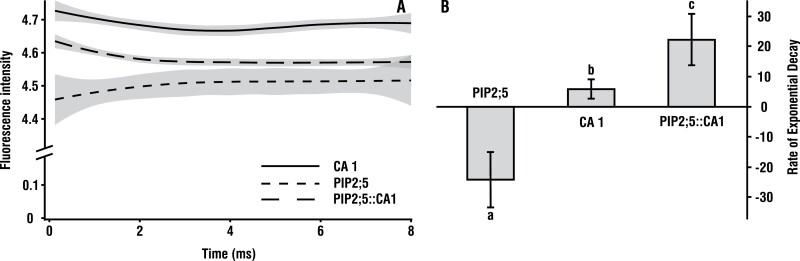
CO_2_ conductance of AtPIP2;5 expressed in yeast. (A) Fluorescence intensity for yeast cells loaded with fluorescein diacetate measured at 0.125 ms intervals. Intracellular acidification in response to the entry of CO_2_ causes a decrease in the fluorescence intensity of yeast cells. Average curves with 95% confidence intervals are presented. (B) CO_2_-induced intracellular acidification rate of *S. cerevisiae* cells expressing *At*PIP2;5, *At*CA1, or both. Yeast cells were exposed in a ratio of 1:1 (v/v) to a CO_2_-mixing buffer (25 mM HEPES, 75 mM NaHCO_3_, pH 6). Kinetics of acidification were measured with an excitation wavelength of 460 nm and emission above 515 nm using a stopped-flow spectrophotometer. Bars represent the CO_2_ permeability of yeast expressed as the exponential decay rate of fluorescence intensity. The kinetics of the decrease in fluorescence were obtained by fitting an exponential decay function to these curves in order to calculate the rate constants. Values are means ±SD of three replicates, and each replicate was comprised of six technical repeats. Different letters denote statistically different values at *P*<0.05.

Yeast cells expressing either *At*PIP2;5, *At*CA1, or both, and loaded with fluorescein diacetate, displayed significantly different fluorescence intensities after the application of the CO_2_ mixing buffer (Fig. 5A). Fluorescence intensity did not decrease in yeast cells expressing only *At*PIP2;5, because *At*CA1 was not present and thus no significant acidification occurred (Fig. 5B). This assay was used as the first control. Yeast cells expressing only *At*CA1 were used as a second control in order to quantify the CO_2_ permeability of the yeast membrane in the absence of *At*PIP2;5. These cells displayed a slight decrease in fluorescence intensity due to CA1-facilitated formation of H_2_CO_3_^–^ and subsequent intracellular acidification (Fig. 5B). When both *At*PIP2;5 and *At*CA1 were expressed together, the fluorescence intensity decreased markedly (Fig. 5B).

### Gene expression

**Fig. 6.**
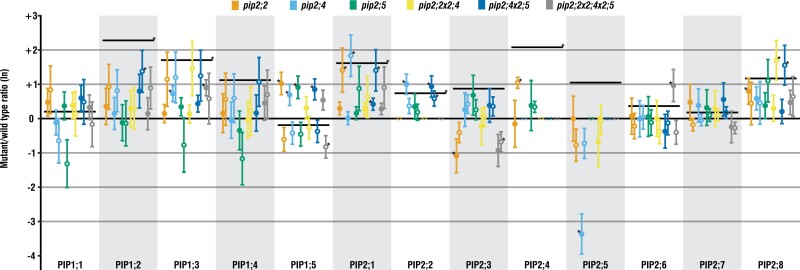
Ratios of *At*PIP expression levels under low and high humidity. Ratios for each mutant line as compared with the respective WT grown under the same conditions are shown. Black lines present the gene expression level of the WT grown under low humidity (LH; filled circles) compared with the WT under high humidity (HH; open circles). Values are means ±SE of 4–6 replicates. Asterisks indicate statistically significant differences compared with the respective WT.

The expression levels of PIP genes (Fig. 6) were generally higher under LH as compared with HH, and this effect was statistically significant for *PIP1;2*, *PIP1;3*, *PIP2;1*, *PIP2;2*, and *PIP2;4*. Under both growing conditions, the knockout mutations generally had no significant effect on the expression of other PIP genes, though there are a few notable exceptions. For example, compared with the WT, in *pip2;2* there was significant up-regulation of *PIP2;1* and *PIP2;4* under HH, whereas under LH, *PIP1;5* and *PIP2;8* were up-regulated and *PIP2;3* down-regulated. In *pip2;4*, compared with the WT, the effect on other PIP genes was small under HH, with only *PIP2;1* significantly up-regulated, but under LH, *PIP1;3*, *PIP1;5*, and *PIP2;2* were up-regulated while the expression of *PIP2;5* was strongly reduced (Fig. 6).

## Discussion

Similarly to earlier reports (Javot *et al.*, 2003; Da Ines *et al.*, 2010; Heckwolf *et al.*, 2011; Wang *et al.*, 2016), the leaf gas exchange of the Arabidopsis plants grown under HH conditions did not appear to be affected by PIP knockout mutations as is apparent from the very uniform values of *A*_net_, *g*_s_, and *E*. Increasing the evaporative demand (higher Vpd) by lowering ambient air humidity increased water flow through the plant (Fig. 2), and hence the contribution of aquaporins to the regulation of water flow through the plant also probably increased, which in part can be seen in the increased PIP expression under LH (Fig. 6). By allowing plants to grow from germination under LH conditions, we aimed to amplify any differences in water relations between the WT and knockout mutants (Fig. 1).

*AtPIP2;5* responds to various stresses such as H_2_O_2_ (Hooijmaijers *et al.*, 2012) and salt (Lee and Zwiazek, 2015), as well as being up-regulated by drought (Alexandersson *et al.*, 2010), but is only expressed at intermediate levels under standard growing conditions, especially in the roots (Jang *et al.*, 2004; Lee *et al.*, 2012). Thus the WT and the *pip2;5* mutant had a similar *A*_net_ and *g*_s_ in our HH environment. However, while *g*_m_ increased in every other genotype grown under LH, *pip2;5* showed no change (Fig. 1E). Furthermore, light–response curves from plants grown under LH (Fig. 4 bottom) showed *A*_net_ and *g*_s_ of this mutant to be significantly less than those of the WT. Soil water deficit commonly decreases *g*_m_ (Warren *et al.*, 2004; Warren, 2008*a*, b) but, since our plants were not experiencing such stress, the inability of *pip2;5* to maintain high *A*_net_ and *g*_m_ led us to conclude that *At*PIP2;5 is involved in the regulation of *g*_m_*in planta*. Our stopped-flow measurements on yeast cells expressing *At*PIP2;5 support this. When *At*PIP2;5 and *At*CA1 were co-expressed, CO_2_ entry into the cells was >100-fold more rapid than in the controls, indicating that the CO_2_ permeability of the membrane was drastically increased by the insertion of *At*PIP2;5. Thus, *At*PIP2;5 is clearly permeable to CO_2_, when expressed in yeast, and its ability to regulate *g*_m_ is likely to be due to it directly facilitating CO_2_ diffusion across the cell membrane. *At*PIP2;5 has not previously been shown to alter CO_2_ fluxes across cell membranes to affect *g*_m_ nor has it, to our knowledge, been tested for CO_2_ permeability.

Up-regulation of *AtPIP2;5* during drought differentiates it from most other PIPs, and the lack of functional *AtPIP2;5* in combination with LH may thus have triggered the drop in *g*_m_. Furthermore, it is only weakly co-expressed with one other PIP isoform, *At*PIP1;4 (Alexandersson *et al.*, 2010), the expression of which was not significantly affected in *pip2;5* (Fig. 6). However, in the very same mutants, the missing interaction of *At*PIP1;4 with *At*PIP2;5 may compromise the proper insertion of *At*PIP1;4 into the plasma membrane. *At*PIP2;5 may thus also be involved in maintaining *g*_m_ across a greater range of watering and humidity regimes by affecting the function of *At*PIP1;4 (Fetter *et al.*, 2004; Zelazny *et al.*, 2007; Otto *et al.*, 2010), which is not only up-regulated by drought (Alexandersson *et al.*, 2010), but has been shown to also contribute to CO_2_ membrane permeability (Li *et al.*, 2015). Further evidence to support this theory is provided by the results of our light–response curves where *g*_s_ was similar for all genotypes, whereas *A*_net_ was clearly lower in *pip2;5* than in the other genotypes. Therefore, contrary to our expectations, *g*_s_ did not behave like *A*_net_ in *pip2;5* in response to a changing light environment, particularly at subsaturating PAR. It would therefore appear that *At*PIP2;5, despite its relatively low abundance (Lee *et al.*, 2012), does reduce resistance to CO_2_ diffusion through the mesophyll under light- and CO_2_-limiting conditions.

Further evidence in support of *At*PIP2;5 regulating *g*_m_ is provided by our *A*–*c*_i_ curves. Its knockout mutation did not appear to significantly affect *J*_max_ or *V*_cmax_, which was not unexpected since none of the PIPs has previously been shown to impact chlorophyll fluorescence or CO_2_ fixation by Rubisco. However, at high [CO_2_], the *c*_i_/*c*_a_ was significantly lower for all mutants lacking *At*PIP2;5 than for the WT (Fig. 3A). Since *c*_a_ was constant, the lower *c*_i_/*c*_a_ was due to lower *c*_i_, probably the result of an incremental effect on a combination of *A*_net_ and *g*_s_ during the *A*–*c*_i_ curve or to an increase in *g*_m_.

*At*PIP2;2 is amongst the most abundantly expressed PIPs in the plant (Javot *et al.*, 2003; Da Ines *et al.*, 2010), but, despite these high levels of expression, its knockout mutation induced no visible phenotype in our study or elsewhere (Javot *et al.*, 2003). However, unlike past reports, we found compensatory up-regulation of other PIP genes (Fig. 6) in mutants lacking functional *AtPIP2;2* grown under both LH and standard HH conditions. Javot *et al.* (2003) found a 14% reduction in the osmotic water conductivity of the root and hypothesized that *At*PIP2;2 plays a crucial role in root water uptake under conditions of low evaporative demand, as in our HH treatment. In our study, two highly expressed genes, *AtPIP2;1* and *AtPIP2;4*, were up-regulated in *pip2;2* rosettes. It is reasonable to assume that these two genes would also have been up-regulated in the roots, potentially explaining why water uptake by the roots as well as flow through the plant was not significantly altered in this mutant. Further support for this assumption is provided by the finding that the root anatomy of *pip2;2* did not differ from that of the WT (Javot *et al.*, 2003).

Interestingly, under LH, *g*_s_ of mutants lacking *At*PIP2;2 did not significantly differ from that of the WT. Although *AtPIP2;2* has previously been shown to be drought sensitive (Alexandersson *et al.*, 2010), our plants were only subjected to LH and in fact we observed quite the opposite. *AtPIP2;2* was significantly, though moderately, up-regulated under LH compared with HH. Furthermore, whole-plant transpiration in *pip2;2* was significantly higher than that of the WT under LH, indicating that water uptake and flow are not impaired by the lack of functional *At*PIP2;2 at the whole-plant level, but may even be overcompensated. Together with the fact that *g*_s_ increased under HH in mutants lacking *At*PIP2;2, this suggests that *At*PIP2;2 may be involved in regulating plant water balance in response to changes in Vpd.

In the LH environment, *pip2;4* had higher *g*_s_ and *E* than the WT. Lack of functional *At*PIP2;4 clearly increases the amount of water lost by the plant (Fig. 2), reducing the plants’ overall water use efficiency. However, this lack of function enabled *pip2;4* plants to maintain higher values of *A*_net_ and *g*_m_ than the WT. Similar effects were found in the double mutants, both of which also lacked functional *At*PIP2;4. This aquaporin is thus a likely candidate for manipulating plant water relations to improve plant carbon gain, though at the expense of water use efficiency.

Since all mutant plants had similar or higher *g*_s_ and *E* than the WT under both growing conditions, this clearly indicates that none of the knockout mutations sufficiently disrupts root water uptake to a degree that could not be compensated for. Thus these mutants did not have a visible phenotype. Furthermore, the lack of significant differences in *g*_min_ (Fig. 3B) or RWC (Supplementary Table S2) suggests that the observed effects on *g*_s_ and *g*_m_ are unlikely to be due to an effect of the knockout mutation on leaf water status. Another factor potentially affecting leaf water status is boundary layer resistance. However, high air circulation decreases this resistance to a negligible minimum under both LH and HH conditions, as well as within the leaf chamber during gas exchange measurements. Therefore, the reported differences between the mutants and the WT are more likely to directly result from the knockout mutation and lack of aquaporin function, rather than being an indirect effect caused by altered plant water status.

We report that *At*PIP2;5 is permeable to CO_2_ when expressed in yeast and that it contributes to the regulation of mesophyll conductance of CO_2_ in leaves of *A. thaliana* under conditions of high evaporative demand. *At*PIP2;4 may play a role in maintaining plant water status, so may also be a suitable target for crop improvement, since the lack of functional *At*PIP2;4 led to a 29% increase in *A*_net_ when VPD was high, though at the expense of water use efficiency. Identification of the mechanisms underpinning these results may represent the means of teasing apart the factors regulating *g*_s_ and *g*_m_.

## Supplementary data

The following supplementary data are available at *JXB* online.

Table S1. Summary of the main environmental variables under high and low humidity.

Table S2. Gas exchange data and variables calculated from the *A*–response curves under high and low humidity.

Table S3. Two-way ANOVA for whole-plant transpiration.

Table S4. Primers used in this study.

Fig. S1. *A*–*c*_c_ curves for all plant lines.

Fig. S2. Fluorescence images of transformed *S. cerevisiae* cells.

Fig. S3. Expression levels of *At*PIP2;5 in *S. cerevisiae*.

erab187_suppl_Supplementary_File001

## Data Availability

The data supporting the findings of this study are available from the corresponding author, David Israel, upon request.

## Abbreviations

*A*_net_: net photosynthetic rate
*A*_sat_: saturating photosynthesis
*c*_a_: atmospheric CO_2_ concentration
CA1: carbonic anhydrase 1
*c*_c_: CO_2_ concentration at the site of carboxylation
*c*_i_: intercellular CO_2_ concentration
*c*_i_*: photorespiratory compensation point
E: whole-plant transpiration
GAMM: generalized additive mixed modelling
*g*_m_: mesophyll conductance of CO_2_
*g*_min_: minimum conductance
*g*_s_: stomatal conductance
HH: high humidity
J: rate of electron transport
*J*_max_: maximum rate of electron transport
LH: low humidity
PAR: photosynthetically active radiation
PIP: plasma membrane intrinsic protein
*R*_d_: respiration in the light
RWC: relative water content
*V*_cmax_: maximum rate of carboxylation
Vpd: vapour pressure deficit
WT: wild type
φ: quantum efficiency
