# Supplementary material for: The contribution of PIP2-type aquaporins to photosynthetic response to increased vapour pressure deficit
Source: J Exp Bot. 2021 Apr 30;72(13):5066–78. doi: 10.1093/jxb/erab187 (PMC8219038; doi:10.1093/jxb/erab187)
Supplement: erab187_suppl_Supplementary_File001 [file erab187_suppl_supplementary_file001.pdf]

Supplementary Material

Table S1: Summary of the main environmental variables under high and low humidity. Plant age refers to the average age at which the plants were measured. Day length was set for the growth room while the greenhouse received the natural photoperiod of sunlight. Due to a non-transparent wall on the west side, the sunset in the greenhouse occurred approximately one hour earlier than the astronomical sunset. All other values are given as daily averages (± SE) and the main differences between the two growing conditions are highlighted in bold.

**Environmental Factor**	**High Humidity**	**Low Humidity**
Average plant age at time of measurement (d)	30.0	31.9
Day length (h)	10 (controlled)	12-13 (astronomical)
PAR (µmol m^{-2} s^{-1})	500 ± 24	690 ± 37
Vpd during the day/night (kPa)	**1.10 ± 0.07 / 0.21 ± 0.07**	**2.24 ± 0.05 / 1.10 ± 0.05**
Relative humidity during the day/night (%)	**51.3 ± 1.8 / 75 ± 1.8**	**22.3 ± 1.3 / 40 ± 1.3**
Air temperature day/night (°C)	25.9/20.0 ± 0.1	25.1/21.0 ± 0.1

Table S2: Gas exchange data and variables calculated from the *A*-response curves under HH and LH conditions. Means and SE are presented with significant differences compared to the WT indicated where the overall effect of genotype in the ANOVA was significant ($p < 0.05$). A_{net} – net photosynthetic rate, c_a – atmospheric CO_2 concentration, c_c – CO_2 concentration at the site of carboxylation, c_i – intercellular CO_2 concentration, *E* – whole-plant transpiration, g_m – mesophyll conductance of CO_2, g_{min} – minimum conductance, g_s – stomatal conductance, HH – high humidity, θ – degree of curvature of a light response curve, J_{max} – maximum rate of electron transport, LCP – light compensation point, LH – low humidity, Q75%A_{sat} – irradiance at which 75% of the saturating rate of photosynthesis is reached, R_d – respiration in the light, RWC – relative water content, V_{cmax} – maximum rate of carboxylation, Vpd – vapour pressure deficit, φ – quantum efficiency, WT – wild type

Variable	WT	*2;2*	*2;4*	*2;5*	*2;2x2;4*	*2;4x2;5*	*2;2x2;4x2;5*
**A_{net} LH ($\mu mol\ m^{-2}\ s^{-1}$)**	10.72 ± 0.78	12.17 ± 1.14	**13.86 ± 1.14 (p = 0.010)**	10.10 ± 1.29	12.36 ± 1.14	**13.06 ± 1.10 (p = 0.041)**	11.56 ± 1.14
**A_{net} HH ($\mu mol\ m^{-2}\ s^{-1}$)**	16.26 ± 1.00	17.18 ± 1.34	15.51 ± 1.34	15.10 ± 1.41	17.17 ± 1.25	17.61 ± 1.22	17.37 ± 1.29
**g_s LH ($mol\ m^{-2}\ s^{-1}$)**	0.124 ± 0.017	0.156 ± 0.026	**0.179 ± 0.026 (p = 0.037)**	0.127 ± 0.029	**0.176 ± 0.026 (p = 0.049)**	**0.193 ± 0.025 (p = 0.008)**	0.156 ± 0.026
**g_s HH ($mol\ m^{-2}\ s^{-1}$)**	0.290 ± 0.034	**0.370 ± 0.046 (p = 0.089)**	0.303 ± 0.046	0.329 ± 0.048	**0.375 ± 0.043 (p = 0.055)**	0.357 ± 0.042	**0.366 ± 0.044 (p = 0.091)**
**g_m LH ($mol\ m^{-2}\ s^{-1}\ bar^{-1}$)**	0.135 ± 0.036	0.145 ± 0.036	0.170 ± 0.043	0.091 ± 0.028	0.179 ± 0.040	0.146 ± 0.043	0.159 ± 0.036
**g_m HH ($mol\ m^{-2}\ s^{-1}\ bar^{-1}$)**	0.118 ± 0.012	0.126 ± 0.012	0.111 ± 0.012	0.107 ± 0.009	0.110 ± 0.011	0.127 ± 0.011	0.131 ± 0.012
**IWUE LH ($\mu mol\ mol^{-1}$)**	89.415 ± 5.693	80.644 ± 8.379	77.316 ± 8.379	83.346 ± 9.440	77.317 ± 8.379	**70.540 ± 8.051 (p = 0.025)**	75.459 ± 8.379
**IWUE HH ($\mu mol\ mol^{-1}$)**	57.671 ± 4.405	46.860 ± 5.910	51.577 ± 5.910	45.994 ± 6.230	47.148 ± 5.522	51.092 ± 5.395	49.164 ± 5.687
**g_{min} ($mmol\ m^{-2}\ s^{-1}$)**	5.55 ± 0.28	5.90 ± 0.41	5.37 ± 0.42	5.75 ± 0.41	5.45 ± 0.40	5.93 ± 0.40	6.29 ± 0.40
**Vpd in cuvette LH (kPa)**	2.296 ± 0.143	2.430 ± 0.210	2.157 ± 0.210	2.586 ± 0.236	2.009 ± 0.210	2.034 ± 0.202	2.331 ± 0.210
**Vpd in cuvette HH (kPa)**	1.201 ± 0.082	1.050 ± 0.109	1.165 ± 0.109	1.092 ± 0.115	1.039 ± 0.102	1.127 ± 0.100	1.071 ± 0.105
**V_{cmax}**	58.75 ± 3.42	56.48 ± 4.33	56.44 ± 4.33	58.04 ± 5.07	52.14 ± 5.41	54.48 ± 5.41	57.05 ± 5.07

**(µmol m^{-2} s^{-1})**							
**J_{max} (µEq m^{-2} s^{-1})**	132.25 ± 6.91	122.90 ± 8.74	122.47 ± 8.74	128.76 ± 10.25	112.89 ± 10.93	122.44 ± 10.93	128.19 ± 10.25
**c_c inflection (µmol)**	529.89 ± 30.61	496.52 ± 38.72	489.46 ± 38.72	506.34 ± 45.40	490.18 ± 48.40	537.99 ± 48.40	537.26 ± 45.40
**c_i/c_a * (µmol µmol^{-1})**	0.82 ± 0.03	0.80 ± 0.03	0.80 ± 0.03	0.81 ± 0.04	0.80 ± 0.04	0.81 ± 0.04	0.82 ± 0.04
**c_i/c_a ** (µmol µmol^{-1})**	0.79 ± 0.01	0.78 ± 0.01	0.78 ± 0.01	**0.75 ± 0.01 (p = 0.008)**	0.77 ± 0.01	**0.75 ± 0.01 (p = 0.013)**	**0.74 ± 0.01 ($p < 0.001$)**
**RWC HH (%)**	93.29 ± 0.54	93.67 ± 0.79	93.95 ± 0.80	93.12 ± 0.79	93.83 ± 0.77	93.20 ± 0.78	94.10 ± 0.77
**Leaf mass area (mg cm^{-2})**	3.279 ± 0.122	3.003 ± 0.178	3.283 ± 0.181	3.080 ± 0.178	3.130 ± 0.173	3.125 ± 0.175	3.054 ± 0.173
**A_{sat} LH (µmol m^{-2} s^{-1})**	21.68 ± 1.09	23.22 ± 1.60	**24.82 ± 1.60 (p = 0.058)**	20.05 ± 1.80	22.80 ± 1.60	23.10 ± 1.54	21.95 ± 1.60
**A_{sat} HH (µmol m^{-2} s^{-1})**	24.17 ± 0.70	**21.07 ± 0.99 (p = 0.003)**	22.36 ± 0.99	22.45 ± 1.01	**20.92 ± 0.99 (p = 0.002)**	**22.08 ± 0.99 (p = 0.038)**	23.21 ± 0.96
**φ LH (mol mol^{-1})**	0.080 ± 0.004	0.078 ± 0.005	0.086 ± 0.005	**0.067 ± 0.006 (p = 0.026)**	0.076 ± 0.005	0.089 ± 0.005	0.079 ± 0.005
**φ HH (mol mol^{-1})**	0.052 ± 0.002	0.052 ± 0.003	0.052 ± 0.003	0.052 ± 0.003	0.051 ± 0.003	0.053 ± 0.003	0.052 ± 0.003
**R_d LH (µmol m^{-2} s^{-1})**	1.47 ± 0.13	1.83 ± 0.19	1.79 ± 0.19	1.81 ± 0.21	1.41 ± 0.19	1.52 ± 0.18	1.45 ± 0.18
**R_d HH (µmol m^{-2} s^{-1})**	2.40 ± 0.09	**2.06 ± 0.13 (p = 0.014)**	2.23 ± 0.17	2.27 ± 0.14	**2.07 ± 0.13 (p = 0.015)**	2.25 ± 0.13	2.32 ± 0.13
**θ LH**	0.564 ± 0.063	0.568 ± 0.092	0.579 ± 0.092	0.551 ± 0.104	0.566 ± 0.092	0.515 ± 0.092	0.660 ± 0.092
**θ HH**	0.767 ± 0.019	0.785 ± 0.026	0.771 ± 0.026	0.759 ± 0.027	0.749 ± 0.026	0.737 ± 0.026	0.780 ± 0.026
**LCP LH (µmol m^{-2} s^{-1})**	18.90 ± 1.62	**24.43 ± 2.39 (p = 0.027)**	21.67 ± 2.39	**28.86 ± 2.69 ($p < 0.001$)**	19.26 ± 2.39	17.77 ± 2.30	18.89 ± 2.39
**LCP HH (µmol m^{-2} s^{-1})**	47.88 ± 2.53	42.58 ± 3.58	44.39 ± 3.58	45.39 ± 3.68	42.57 ± 3.58	44.39 ± 3.58	46.83 ± 3.50
**Q75%A_{sat} LH (µmol m^{-2} s^{-1})**	652.57 ± 62.98	743.50 ± 92.70	703.37 ± 92.70	844.03 ± 104.44	709.22 ± 92.70	729.67 ± 89.06	565.59 ± 92.70
**Q75%A_{sat} LH (µmol m^{-2} s^{-1})**	929.81 ± 53.72	784.26 ± 75.97	848.39 ± 75.97	883.68 ± 78.05	854.54 ± 75.97	915.76 ± 75.97	877.50 ± 74.23

**E LH (ml cm^{-2})**	1.15 ± 0.07	**1.41 ± 0.09 (*p* = 0.005)**	**1.38 ± 0.09 (*p* = 0.014)**	1.11 ± 0.09	1.26 ± 0.09	1.14 ± 0.10	1.29 ± 0.10
**E HH (ml cm^{-2})**	0.37 ± 0.04	0.38 ± 0.06	0.38 ± 0.06	0.37 ± 0.06	0.36 ± 0.06	0.37 ± 0.06	0.37 ± 0.06
**Final leaf area LH (cm^{-2})**	12.6 ± 1.7	13.5 ± 2.3	16.1 ± 2.4	15.2 ± 2.3	14.1 ± 2.3	14.5 ± 2.4	11.4 ± 2.4
**Final leaf area HH (cm^{-2})**	37.4 ± 1.1	**33.4 ± 1.5 (*p* = 0.011)**	**31.1 ± 1.6 (*p* < 0.001)**	**34.0 ± 1.5 (*p* = 0.031)**	**33.8 ± 1.5 (*p* = 0.021)**	35.9 ± 1.5	36.1 ± 1.5

c_i/c_a **low CO_2 end of A - c_i curve; c_i/c_a *** high CO_2 end of A - c_i curve.

Table S3: Two-Way-ANOVA tables for whole-plant transpiration and leaf area

**Whole-plant transpiration in terms of cumulative leaf area**				
	Sum of Squares	Df	F-value	*p*-value
Line	0.611	6	3.588	**0.002**
Treatment	37.139	1	11308.689	< **0.001**
Line:Treatment	0.614	6	3.606	**0.002**
Residuals	5.080	179		
	Estimate	Std. Error	*t*-value	*p*-value
WT (Intercept)	0.370	0.043	8.508	< **0.001**
pip2;2	0.379	0.061	0.141	0.888
pip2;4	0.379	0.063	0.144	0.885
pip2;5	0.371	0.062	0.012	0.991
pip2;2x2;4	0.356	0.062	-0.230	0.818
pip2;4x2;5	0.365	0.062	-0.075	0.940
pip2;2x2;4x2;5	0.371	0.062	0.018	0.986
WT (Treatment LH)	1.150	0.069	11.339	< **0.001**
pip2;2:Treatment LH	1.414	0.092	2.858	**0.005**
pip2;4:Treatment LH	1.385	0.095	2.484	**0.014**
pip2;5:Treatment LH	1.111	0.093	-0.424	0.672
pip2;2x2;4:Treatment LH	1.264	0.092	1.239	0.217
pip2;4x2;5:Treatment LH	1.137	0.096	-0.131	0.896
pip2;2x2;4x2;5:Treatment LH	1.286	0.096	1.419	0.158
**Total leaf area at 30 days**				
	Sum of Squares	Df	F-value	*p*-value
Line	424.6	6	3.948	< **0.001**
Treatment	26068.2	1	11454.259	< **0.001**
Line:Treatment	98.3	6	0.914	0.486
Residuals	3208.7	179		
	Estimate	Std. Error	*t*-value	*p*-value
WT (Intercept)	37.355	1.093	34.171	< **0.001**
pip2;2	33.423	1.522	-2.584	**0.011**
pip2;4	31.060	1.573	-4.001	< **0.001**
pip2;5	33.989	1.546	-2.177	**0.031**
pip2;2x2;4	33.753	1.546	-2.330	**0.021**
pip2;4x2;5	35.866	1.546	-0.963	0.337
pip2;2x2;4x2;5	36.052	1.546	-0.843	0.400
WT (Treatment LH)	12.615	1.729	-14.313	< **0.001**
pip2;2:Treatment LH	13.454	2.321	0.362	0.718
pip2;4:Treatment LH	16.114	2.376	1.473	0.143
pip2;5:Treatment LH	15.224	2.337	1.116	0.266
pip2;2x2;4:Treatment LH	14.119	2.319	0.649	0.517
pip2;4x2;5:Treatment LH	14.469	2.411	0.769	0.443
pip2;2x2;4x2;5:Treatment LH	11.422	2.411	-0.495	0.622

Table S4: Primers used in this study

Yeast Transformation	
**Gene**	**Primer**
AtßCA1	For: 5´-GGGGACAAGTTTGTACAAAAAAGCAGGCTTCATGGGAACCGAAGCATAC-3´
AtßCA1	Rev: 5´-GGGGACCACTTTGTACAAGAAAGCTGGGTCTACAGAGCTAGTTTCGGA-3´
GTAtPIP2-5	For: 5´-GGGGACAAGTTTGTACAAAAAAGCAGGCTTCATGACGAAGGAAGTGGTTGG-3´
GT AtPIP2-5	Rev: 5´ -GGGGACCACTTTGTACAAGAAAGCTGGGTCTTAAACGTGAGGCTGGCTCC-3´
AtPIP2-5qPCR	For: 5´- TCGCATGGGCCTTTGG-3´
AtPIP2-5qPCR	Rev: 5´- GCCGGCGGTGCAGTAG-3´
AtβCA1qPCR	For: 5´- CAATGTGGCCGATGTGAAAG-3´
AtβCA1qPCR	Rev: 5´- CAGAGCTAGTTTCGGAGAG-3´
ScACT1	For: 5´- AGAGTTGCCCCAGAAGAACA-3´
ScACT1	Rev: 5´- GGCTTGGATGGAAACGTAGA-3´
**Gene Expression**	
**PIP Gene**	**Primer**
PIP1;1 (At3g61430)	For: 5´- TCTTAACCCAAAGGCCAACA-3´
PIP1;1 (At3g61430)	Rev: 5´- CAGAATCCAGCACAATACCG-3´
PIP1;2 (At2g45960)	For: 5´- TGGGATGACCACTGGGTGTTT-3´
PIP1;2 (At2g45960)	Rev: 5´- GGGATGGCTCTGATGACAACC-3´
PIP1;3 (At1g01620)	For: 5´- CTTCCGTCGGAATCCAAGGT-3´
PIP1;3 (At1g01620)	Rev: 5´- CGTGTGAGCGAAAGCTTCCT-3´
PIP1;4 (At4g00430)	For: 5´- GGAATCTCTGGTGGGCACAT-3´
PIP1;4 (At4g00430)	Rev: 5´- CTCCGAGACATTGCATCACG-3´
PIP1;5 (At4g23400)	For: 5´- TGGGATGACCATTGGATTTT-3´
PIP1;5 (At4g23400)	Rev: 5´- TCTGGACCGTGGAATCTTTC-3´
PIP2;1 (At3g53420)	For: 5´- GCTGGAATCTCAGGAGGACATATT-3´
PIP2;1 (At3g53420)	Rev: 5´- AGCTCCAAGGCACTGCATTACT-3´
PIP2;2 (At2g37170)	For: 5´- TAGATTGCGGCGGAGTTGGA-3´
PIP2;2 (At2g37170)	Rev: 5´- CGCTGGGTTAATGTGACCACCA-3´
PIP2;3 (At2g37180)	For: 5´- CAAAGACGTGGAAGGACCTGAG-3´

PIP2;3 (At2g37180)	Rev: 5´- ACTTGGTAAGCTCGTCCGCA-3´
PIP2;4 (At5g60660)	For: 5´- TTCGACGCAGAGGAGCTTAC-3´
PIP2;4 (At5g60660)	Rev: 5´- GCTACGAACTCGGCGATGACT-3´
PIP2;5 (At3g54820)	For: 5´- CCGATGGCTACAACAAAGGT-3´
PIP2;5 (At3g54820)	Rev: 5´- CACGTGAGAGTCACGAGCAT-3´
PIP2;6 (At2g39010)	For: 5´- TGGTGGGCATATTAATCCGGCAGT-3´
PIP2;6 (At2g39010)	Rev: 5´- TGACCAAAGCCACACCACAAATGG-3´
PIP2;7 (At4g35100)	For: 5´- GGCATCTCTGGTGGACACATC-3´
PIP2;7 (At4g35100)	Rev: 5´- CAACTCCACAAGTGGCTCCG-3´
PIP2;8 (At2g16850)	For: 5´- CGTGGGATGACCAATGGATC-3´
PIP2;8 (At2g16850)	Rev: 5´- TGCGTTGCTTCGGAACGAG-3´
**Reference Gene**	
YLS8 (At5g08290)	For: 5´ - TTACTGTTTCGGTTGTTCTCCATTT-3´
YLS8 (At5g08290)	Rev: 5´ - CACTGAATCATGTTCGAAGCAAGT-3´
MON1 (At2g28390)	For: 5´ - CAGACAAGGCGATGGCGATA-3´
MON1 (At2g28390)	Rev: 5´ - GCTTTCTCTCAAGGGTTTCTGGGT-3´
TIP41 (At4g34270)	For: 5´ - GTGAAAACTGTTGGAGAGAAGCAA-3´
TIP41 (At4g34270)	Rev: 5´ - TCAACTGGATACCCTTTCGCA-3´

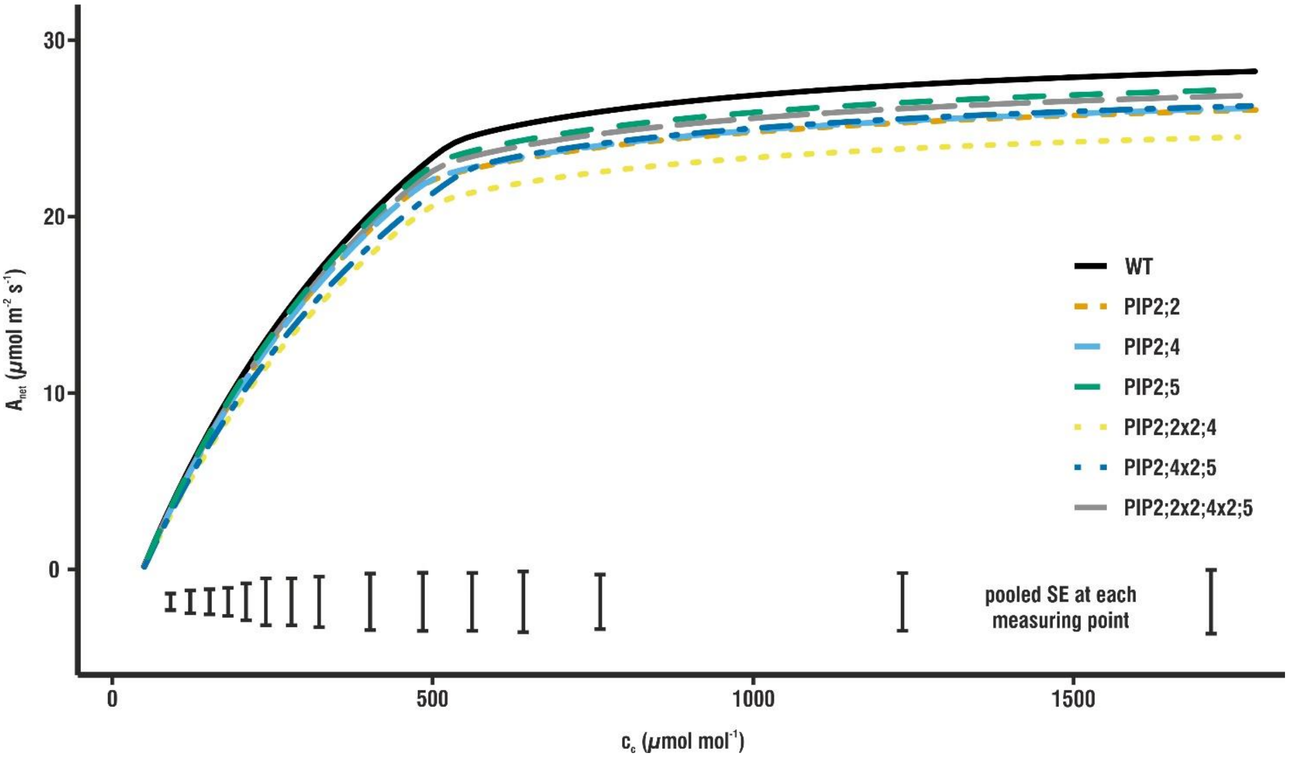

Figure S1: Fitted A-c_c curves for all mutant lines and the WT measured at PAR = 1500 µmol m^{-2} s^{-1}. At ambient CO_2 (≈ 400 µmol mol^{-1} air), A_{net} is CO_2-limited, because this CO_2 concentration is situated in the linear part of the curve. Fitted means with the pooled SE are shown at the bottom of the graph (n = 8-10).

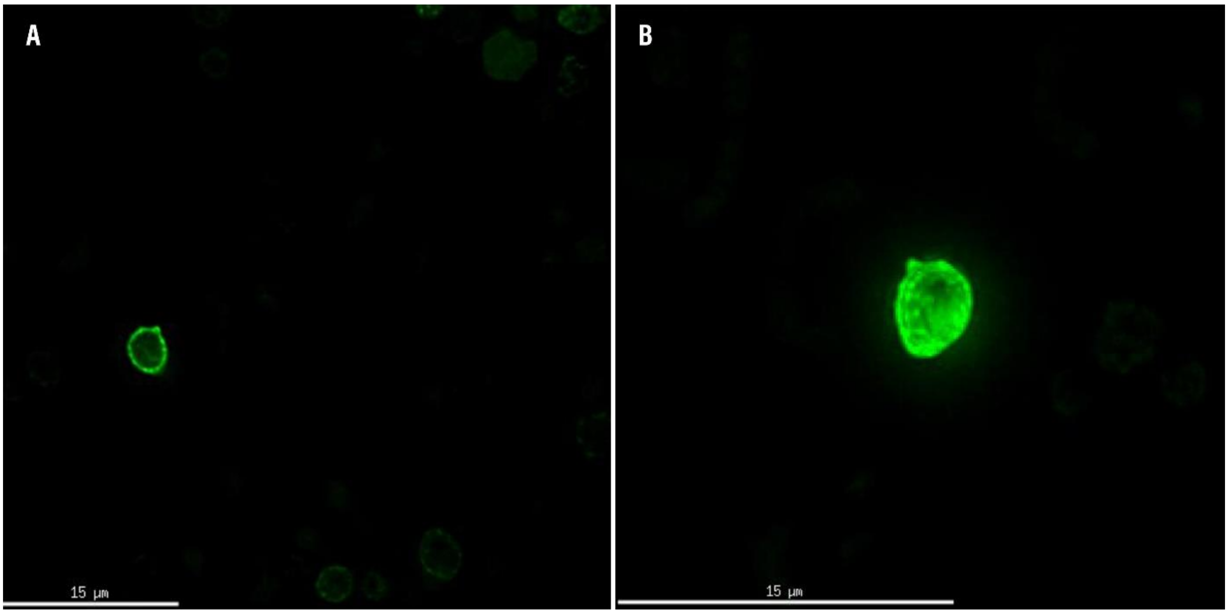

Figure S2: Representative fluorescence microscopy images (**A** – 2D; **B** – 3D) of transformed *S. cerevisiae* cells expressing *At*PIP2;5 with a C-terminal eGFP tag. The protein clearly localises to the plasma membrane.

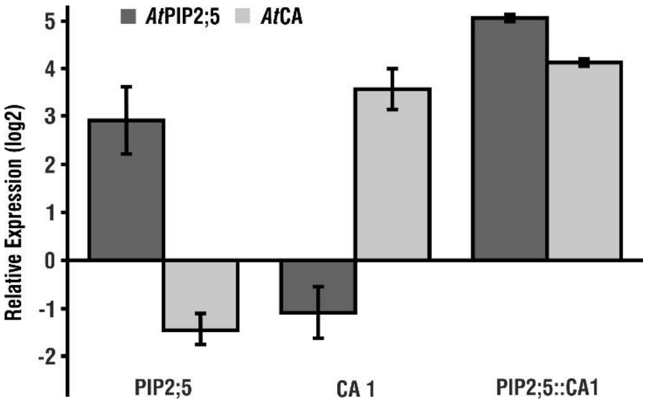

Figure S3: Relative expression of *At*PIP2;5 and *At*CA1 in transformed *S. cerevisiae* cells expressing *At*PIP2;5, *At*CA1 or both. The yeast strains are indicated at the bottom of the graph. Values are means ± SD.
